# Understanding mechanisms of impact from community-led delivery of HIV self-testing: Mediation analysis of a cluster-randomised trial in Malawi

**DOI:** 10.1371/journal.pgph.0001129

**Published:** 2022-10-27

**Authors:** Pitchaya P. Indravudh, Fern Terris-Prestholt, Melissa Neuman, Moses K. Kumwenda, Richard Chilongosi, Cheryl C. Johnson, Karin Hatzold, Elizabeth L. Corbett, Katherine Fielding

**Affiliations:** 1 Department of Global Health and Development, London School of Hygiene & Tropical Medicine, London, United Kingdom; 2 Malawi-Liverpool-Wellcome Trust Clinical Research Programme, Blantyre, Malawi; 3 Joint United Nations Programme on HIV/AIDS, Geneva, Switzerland; 4 Department of Infectious Disease Epidemiology, London School of Hygiene & Tropical Medicine, London, United Kingdom; 5 Population Services International Malawi, Lilongwe, Malawi; 6 Global HIV, Hepatitis and Sexually Transmitted Infections Programmes, World Health Organisation, Geneva, Switzerland; 7 Department of Clinical Research, London School of Hygiene & Tropical Medicine, London, United Kingdom; 8 Population Services International, Washington, District of Columbia, United States of America; 9 School of Public Health, University of the Witwatersrand, Johannesburg, South Africa; University of California San Francisco, UNITED STATES

## Abstract

Community HIV strategies are important for early diagnosis and treatment, with new self-care technologies expanding the types of services that can be led by communities. We evaluated mechanisms underlying the impact of community-led delivery of HIV self-testing (HIVST) using mediation analysis. We conducted a cluster-randomised trial allocating 30 group village heads and their catchment areas to the community-led HIVST intervention in addition to the standard of care (SOC) or the SOC alone. The intervention used participatory approaches to engage established community health groups to lead the design and implementation of HIVST campaigns. Potential mediators (individual perceptions of social cohesion, shared HIV concern, critical consciousness, community HIV stigma) and the outcome (HIV testing in the last 3 months) were measured through a post-intervention survey. Analysis used regression-based models to test (i) intervention-mediator effects, (ii) mediator-outcome effects, and (iii) direct and indirect effects. The survey included 972 and 924 participants in the community-led HIVST and SOC clusters, respectively. The community-led HIVST intervention increased uptake of recent HIV testing, with no evidence of indirect effects from changes in hypothesised mediators. However, standardised scores for community cohesion (adjusted mean difference [MD] 0.15, 95% CI -0.03 to 0.32, p = 0.10) and shared concern for HIV (adjusted MD 0.13, 95% CI -0.02 to 0.29, p = 0.09) were slightly higher in the community-led HIVST arm than the SOC arm. Social cohesion, community concern, and critical consciousness also apparently had a quadratic association with recent testing in the community-led HIVST arm, with a positive relationship indicated at lower ranges of each score. We found no evidence of intervention effects on community HIV stigma and its association with recent testing. We conclude that the intervention effect mostly operated directly through community-driven service delivery of a novel HIV technology rather than through intermediate effects on perceived community mobilisation and HIV stigma.

## Introduction

Knowledge of HIV status is critical for controlling HIV transmission, with 1.7 million people newly infected with HIV in 2018 [1]. Effective HIV testing services (HTS) can enable early diagnosis and linkage to treatment among HIV-positive individuals and linkage to prevention among individuals at risk for HIV. Expanded HTS provision through health facilities has improved awareness of HIV status in sub-Saharan Africa, which contributes the majority of new HIV cases [1]. Community HIV strategies can facilitate early diagnosis and treatment [2, 3] to reduce HIV-related morbidity and mortality and limit HIV transmission through treatment and prevention [4]. Self-care products, including HIV self-testing (HIVST), are also generating opportunities beyond health facilities to reach underserved population sub-groups [5, 6].

Community-led strategies for HIV prevention and management involve community groups or organisations leading the design and implementation of HIV programmes [7], with new self-care technologies expanding the types of interventions that can be led by communities. Previous studies have reported improved identification of HIV-positive cases and reduced HIV incidence when communities were involved in the provision of mobile HTS [8, 9]. Community mobilisation approaches that address social and structural drivers of HIV can also impact protective HIV behaviours, including improved condom use and reduced concurrency of sexual partners [10]. Across disease areas, studies have demonstrated the health impact of interventions involving community participation [11–13]. Understanding how community-led interventions affect outcomes is important for maximising the effect and cost-efficiency of community health programmes, though evidence on their pathways to impact is limited [7].

Mediation analysis involves evaluating how an intervention changes an outcome by testing hypotheses about the potential causal mechanisms [14]. A mediator is an intermediate variable that is affected by an exposure and subsequently affects an outcome, with statistical techniques used to quantify the mediation effect [15]. Mediation analysis has been applied within randomised trials to test hypothesised pathways underlying the intervention effect on an outcome [14]. Findings from mediation analysis can therefore support explanation of cause-effect relationships and inform optimisation of interventions to influence key mechanisms.

In the current study, we assessed mediation within a cluster-randomised trial of community-led delivery of HIVST in Malawi. Primary analysis from the trial previously reported an increase in the proportion of the population who tested for HIV, including adolescents 15–19 years, older adults 40 years and above, and men [16]. We examined whether changes in the hypothesised mediators, community mobilisation domains and community HIV stigma, mediated the impact of the intervention on HIV testing, aiming to consider broader lessons for community-led programmes. Specifically, we tested (i) the effect of the intervention on the potential mediators, (ii) the effect of the potential mediators on recent HIV testing, and (iii) the direct intervention effect on recent testing and the indirect effect from changing the potential mediators.

## Materials and methods

### Ethics statement

The trial, which is registered with ClinicalTrials.gov (NCT03541382), was conducted as part of the Unitaid/PSI HIV Self-Testing Africa Initiative (STAR) [http://hivstar.lshtm.ac.uk/]. Ethical approvals were received from the University of Malawi College of Medicine (P.01/18/2332), London School of Hygiene & Tropical Medicine (14761), and WHO (STAR-comm led CRT-Malawi).

### Trial design, procedures, and data collection

We evaluated the role of community mobilisation domains and community HIV stigma as mediators between community-led delivery of HIVST and recent HIV testing within a cluster-randomised trial [17]. The trial was conducted in Mangochi district and randomised 30 group village heads and their catchment areas 1:1 to the community-led HIVST intervention in addition to the standard of care (SOC) or the SOC alone. The community-led HIVST intervention used participatory approaches to engage established community health groups to lead the design and implementation of HIVST campaigns [18]. Community actors included community health action groups and community volunteers, who respectively provide community health services at group village head and village-level, and government community health workers (CHW). The SOC included HIV testing by lay counsellors through government health facilities and periodic community-based outreach. The study team included Population Services International (PSI) Malawi, the Malawi-Liverpool-Wellcome Trust Clinical Research Programme, and the Ministry of Health.

The intervention adapted participatory learning and action methods, with each cluster developing HIVST campaign strategies unique to their respective areas. Implementation was staggered in groups of two-to-three clusters. The study team held two-day participatory workshops attended by community health action groups and CHWs. In their respective clusters, participants defined determinants of HIV infection, mapped HIV services and barriers to access, and identified priority sub-groups with low uptake of HIV services. Participants designed cluster-specific HIVST campaigns and decided on how to distribute HIVST kits, provide support for linkage to routine HIV care, and generate demand for HIVST. The study team then conducted two-day trainings with community volunteers on supporting use and interpretation of HIVST kits and linkage to HIV prevention and treatment, communicating HIV prevention messages, managing social harms, handling and storing kits, and collecting data. Afterwards, community health actions groups, community volunteers, and CHWs led a fixed seven-day campaign based on strategies developed for each cluster. Cluster residents aged 15 years and older were eligible to take an HIVST kit for themselves and for secondary distribution. The study team provided the OraQuick HIV Self-Test (Orasure Technologies, Thailand), instructional materials, data collection tools, and nationally standardised gratuity of MWK 7,000 (US$10) per volunteer.

Outcomes were measured through a post-intervention survey administered eight-to-12 weeks after the start of the intervention in community-led HIVST clusters or matched dates in SOC clusters. In each cluster, villages with at least 500 residents and located near the group head village were randomly selected, with households recruited using a clockwise spiral from a defined location. The survey aimed to recruit at least 250 participants based on sample size calculations for the trial, with cluster residents aged 15 years and older eligible. Cluster residents provided written informed consent or assent with parent or guardian consent for adolescents 15–17 years. Participants were interviewed on their sociodemographic background and prior HIV-related experience. Process data were collected through the survey and HIVST registers.

### Mediation framework

**Fig 1:**
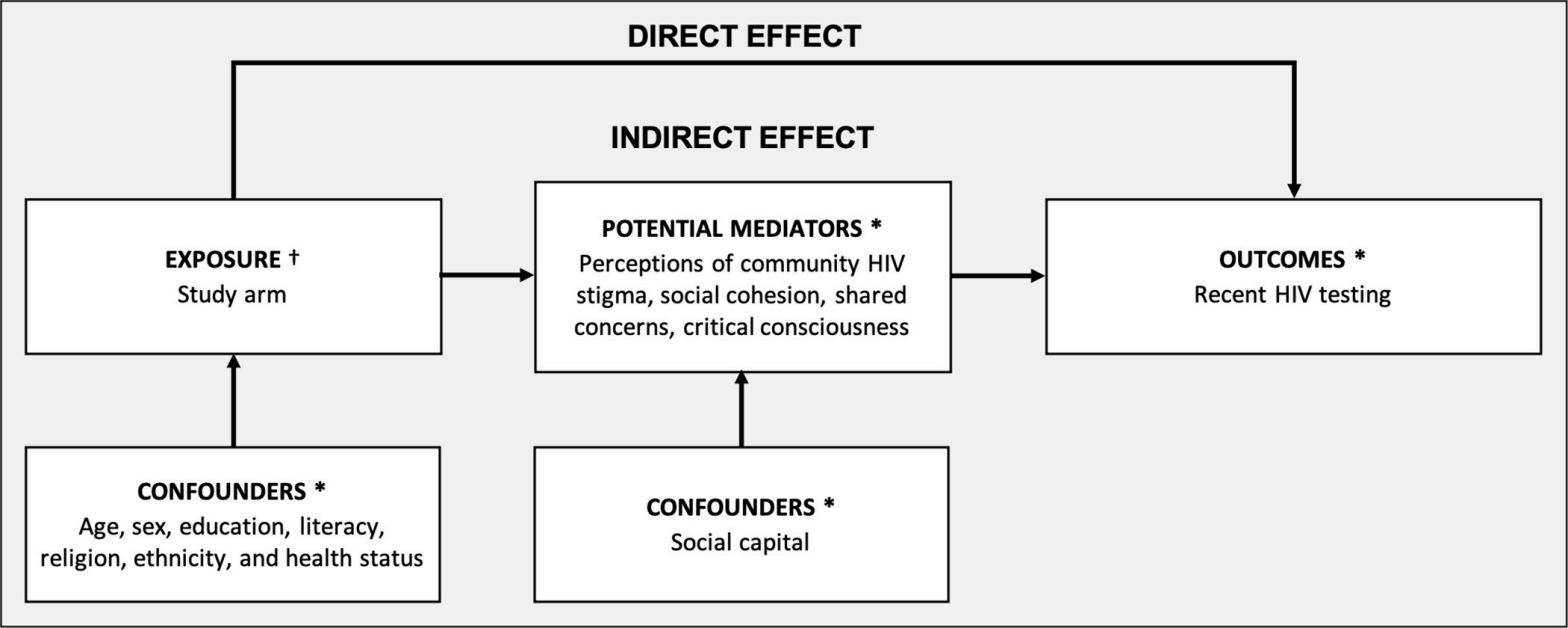
Diagram of mediation framework. HIVST, HIV self-testing. Causal directed acrylic graph of the mediation framework. * Measured at the individual level. † Measured at the cluster level.

The causal directed acyclic graph illustrating the mediation framework for the current study is presented in Fig 1. Potential mediators were identified based on a conceptual framework drawn from the literature on community participation in health interventions. Community participation can be conceptualised along a continuum of increasing empowerment [18], defined as “a social action process by which individuals, communities, and organisations gain mastery over their lives in the context of changing their social and political environment to improve equity and quality of life” [19]. Most practice of community empowerment for health is operationalised through participatory learning and action methods that engage community groups and organisations in the design, implementation, and evaluation of health interventions [18]. Localising decision-making and resource allocation is posited to enhance the coverage and efficiency of health interventions, while devolvement of power and control to marginalised populations is proposed to enable more equitable healthcare distribution [20].

In the context of HIV prevention, Lippman (2013) proposed multiple domains of community mobilisation that would need to be affected to improve HIV-related behaviours and outcomes [21]. Building social cohesion, specifically through shared trust or a common sense of identity, was regarded as a necessary antecedent for successful social mobilisation [21, 22]. Raising critical consciousness through collective dialogue and action was also considered an important component of community mobilisation [21–23]. Additional domains included shared concern for HIV as a priority health issue, participation in collective action, organisational structures and networks facilitating collective action, and leadership [21, 23].

Another hypothesised mechanism of action is by influencing HIV stigma, which has been consistently noted as a barrier to engagement with HIV services [24, 25]. HIV stigma stems from drivers such as fear of HIV infection and social judgement and can subsequently impede access and utilisation of HIV services [26]. Community-led strategies could change norms around care-seeking by activating community support for HIV prevention and treatment. A separate hypothesis suggests the role of HIVST in reducing HIV stigma by empowering individuals and normalising HIV testing [27].

For the current study, we hypothesised that individual-level community mobilisation domains and community HIV stigma acted as mediators between the community-led HIVST intervention and the outcome of tested for HIV in the last 3 months. We collected data on hypothesised mediators in the post-intervention survey among a random sample (approximately 20%) of participants receiving an extended questionnaire. Community constructs are commonly captured at the individual level to represent individual perceptions within the community or aggregated at the community level to denote shared perceptions [28]. Given the brief implementation period, we hypothesised that the intervention would likely impact individual perceptions of community measures rather than broader norms. To measure dimensions of community mobilisation, we used a subset of domains from previously validated scores [28]. Data were captured on perceived social cohesion, a six-item scale for sense of community; perceived shared HIV concern, a 10-item scale for concern related to HIV prevention; and perceived critical consciousness, an 11-item scale for collective problem assessment and resolution (Table A in S1 Text) [28]. Community HIV stigma included five items measuring perceptions of HIV stigma within the community, with responses based on a three-point Likert scale (Table A in S1 Text) [29].

### Statistical analysis

Analysis was restricted to participants providing complete data for the outcome and potential mediators. We assessed implementation outcomes, including awareness and uptake of HIV self-testing, and evaluated intervention and mediation effects. Our mediation model estimated the effect of the cluster-level intervention (community-led HIVST) on the individual-level mediators (social cohesion, shared HIV concern, critical consciousness, community HIV stigma) and outcome (tested for HIV in the last 3 months). Individual-level scores for each potential mediator were generated by summing the question items and standardising the raw scores, with higher scores representing higher levels of each domain. To assess scale reliability, we calculated Raykov’s rho from confirmatory factor analysis using a weighted least squares approach [30]. Coefficients for social cohesion (0.86), shared concern (0.95), critical consciousness (0.96), and community HIV stigma (0.77) showed acceptable reliability.

Mediation analysis was based on a counterfactual framework that extends the product-of-coefficients approach to accommodate a common binary outcome and interaction between the intervention and mediator [15, 31–33]. Effect estimates include natural direct and indirect effects. The direct effect is the intervention effect on the outcome if the mediator was fixed at the level it would have taken in the absence of the intervention. The indirect effect measures the mediation effect; that is, the effect on the outcome caused by the intervention effect on the mediator and the subsequent effect of the mediator on the outcome. Effects can be causally interpreted assuming control is made for intervention-mediator, intervention-outcome, and mediator-outcome confounding and mediator-outcome confounders are not affected by the intervention [15]. Randomisation of the intervention can minimise confounding bias, though further control may be needed to account for cluster randomisation [34]. Adjustment for mediator-outcome confounding is also important given the strong assumptions required for causal interpretation of direct and indirect effects.

We fitted a set of regression models for each potential mediator. To estimate intervention-mediator effects, model 1 included linear regression of the potential mediator on the study arm. The model also included a set of covariates that showed imbalance between study arms (sex, age group, literacy, religion, ethnicity, health status), or was a potential mediator-outcome confounder (social capital) as identified through Fig 1. Social capital, defined as membership in community groups, was selected since the measure represented a time-invariant measure of social relationships and networks (Table A in S1 Text). A random effect for the cluster was used to account for the cluster-randomised design [34, 35]. To estimate mediator-outcome effects, we used a Poisson model with robust standard errors to approximate risk ratios, since the outcome was common [36]. Model 2, which was stratified by arm, used Poisson regression and included the outcome on the mediator, covariates, and the mediator-outcome confounder. We investigated the relationship between the standardised score of the mediator and the outcome by including linear and quadratic terms of the mediator. The model also adjusted for clustering with a random effect.

To calculate direct and indirect effects, we used estimates from Model 1 and a third model [15]. Model 3 included Poisson regression of the outcome on the study arm, the potential mediator, an intervention-mediator interaction term, covariates, and the mediator-outcome confounder, with a robust standard error and random effect for cluster. Mediators showing a non-linear relationship with the outcome were log-transformed in both models. To calculate confidence intervals for direct and indirect effects, we used a bias-corrected bootstrap approach with 1,000 replicates [37]. To explore heterogeneity in intervention and mediation effects, we additionally stratified our analysis by sex and age group, with a focus on adolescents 15–19 years and older adults 40 years and above due to more substantial gaps in undiagnosed HIV. Stata version 14.0 was used for statistical analysis.

## Results

Response rates for the post-intervention survey were 90.2% (3960/4388) and 89.2% (3920/4394) in the community-led HIVST and SOC arms, respectively (Fig A in S1 Text). Of eligible participants, 24.8% (1955/7880) were selected for the extended module. Most participants were included in the primary analysis, with 97.0% (970/1000) in the community-led HIVST arm and 96.6% (923/955) in the SOC arm providing complete data. The majority of participants obtained primary-level education or below and were married (Table 1). Individual characteristics were mainly balanced between arms.

**Table 1:** Comparison of population characteristics by study arm.

	Community-led HIVST	SOC
	n (%)	n (%)
**Household characteristics**	(N = 834)	(N = 822)
Adults (median/range)*	2 (1–8)	2 (0–10)
Children (median/range)*	1 (0–1)	1 (0–1)
Household wealth index †		
Lowest	177 (22.8%)	174 (22.7%)
Second	157 (20.2%)	174 (22.7%)
Third	157 (20.2%)	150 (19.6%)
Fourth	131 (16.9%)	137 (17.9%)
Highest	154 (19.8%)	130 (17.0%)
**Individual characteristics**	(N = 970)	(N = 923)
Male	394 (40.6%)	363 (39.3%)
Age (median/range)	29 (15–96)	29 (15–90)
Age group		
15–19 years	214 (22.1%)	193 (20.9%)
20–39 years	478 (49.3%)	476 (51.6%)
≥ 40 years	278 (28.7%)	254 (27.5%)
Marital status		
Married or living together	609 (62.8%)	581 (62.9%)
Separated, divorced or widowed	150 (15.5%)	125 (13.5%)
Never married	211 (21.8%)	217 (23.5%)
Educational attainment		
None	414 (42.7%)	396 (42.9%)
Primary	457 (47.1%)	442 (47.9%)
Secondary or higher	99 (10.2%)	85 (9.2%)
Literate	562 (57.9%)	515 (55.8%)
Muslim	699 (72.1%)	695 (75.3%)
Ethnicity		
Yao	688 (70.9%)	681 (73.8%)
Ngoni	122 (12.6%)	103 (11.2%)
Other	160 (16.5%)	139 (15.1%)
Self-rated health status		
Very good	394 (40.6%)	318 (34.5%)
Good	403 (41.5%)	425 (46.0%)
Fair	80 (8.2%)	83 (9.0%)
Poor	93 (9.6%)	97 (10.5%)

HIVST, HIV self-testing; SOC, standard of care.

* 13 missing values in the HIVST arm and 6 missing values in the SOC arm.

† 58 missing values in the HIVST arm and 57 missing values in the SOC arm.

### Implementation

The community-led HIVST intervention was delivered in 15 eligible clusters from 5th October, 2018 to 17th January, 2019. HIVST campaigns were implemented by 157 community health action group members (cluster mean 10.5) and 190 community volunteers (cluster mean 12.7; Table B in S1 Text). Implementation strategies involved sensitisation and distribution of HIVST kits at village head-led community meetings, homes, and fixed locations and social hotspots, including schools, churches and mosques, boreholes, fishing docks, sports fields, and video shows. Strategies to support linkage to routine HIV services included active post-test follow-up, phone referrals to health facilities, and material assistance such as transportation funds. Overall, 24,316 HIVST kits (cluster mean 1621) were distributed.

Self-testing for HIV in the last 3 months was 72.6% (704/970) in the community-led HIVST arm, ranging by cluster from 40.3% to 92.7%, and 5.4% (50/923) in the SOC arm (Table B in S1 Text). In the community-led HIVST arm, HIVST uptake was lowest among women aged 40 years and above (65.2%, 101/155) and highest among women aged 20 to 39 years (82.5%, 241/292; Fig B in S1 Text). The proportion of participants who had heard of HIVST was 96.1% (932/970) in the community-led HIVST arm, varying by cluster from 83.5% to 100.0%, and 36.5% (337/923) in the SOC arm.

### Effect of the intervention on potential mediators

Table 2 includes estimates of the intervention effect on standardised scores for the potential mediators. Compared with the SOC arm, social cohesion (adjusted mean difference [MD] 0.15, 95% CI -0.03 to 0.32, p = 0.10) and shared concern for HIV (adjusted MD 0.13, 95% CI -0.02 to 0.29, p = 0.09) were slightly higher in the community-led HIVST arm, though evidence of an intervention effect was weak. Evidence of differences between study arms was not observed for community HIV stigma (adjusted MD -0.01, 95% CI -0.18 to 0.16, p = 0.91) and critical consciousness (adjusted MD 0.11, 95% CI -0.08 to 0.31, p = 0.26).

**Table 2:** Effect of community-led HIV self-testing intervention and potential mediators.

		(1)	(2)		
		Effect of intervention on potential mediator *	Effect of potential mediator on HIV testing in the last 3 months by study arm †		
			Community-led HIVST	SOC	p-value for interaction for study arm ‡
		Adjusted mean difference (95% CI)	Adjusted risk ratio (95% CI)	Adjusted risk ratio (95% CI)	
		p-value	p-value	p-value	
(A)	Community HIV stigma	-0.01 (-0.18, 0.16)	0.97 (0.93, 1.01)	0.91 (0.82, 1.02)	0.42
		0.91	0.12	0.10	
(B)	Social cohesion	0.15 (-0.03, 0.32)	0.92 (0.87, 0.98)	1.02 (0.96, 1.08)	0.12
		0.10	0.006	0.59	
	Social cohesion^2^		0.95 (0.91, 0.99)	1.00 (0.94, 1.06)	
			0.01	0.94	
(C)	Shared concern for HIV	0.13 (-0.02, 0.29)	0.91 (0.87, 0.97)	0.98 (0.92, 1.04)	0.18
		0.09	0.001	0.47	
	Shared concern for HIV^2^		0.94 (0.91, 0.98)	0.98 (0.94, 1.02)	
			0.006	0.42	
(D)	Critical consciousness	0.11 (-0.08, 0.31)	0.92 (0.86, 0.99)	1.02 (0.92, 1.13)	0.09
		0.26	0.02	0.69	
	Critical consciousness^2^		0.96 (0.92, 1.01)	1.01 (0.92, 1.10)	
			0.10	0.88	

C, control; HIVST, HIV self-testing; I, intervention; SOC, standard of care. N = 1893.

* Adjusted mean difference for the study arm (I-C). Model 1 is a linear regression model of the potential mediators on the study arm, with each mediator evaluated separately as the outcome in Models A to D. Analysis adjusts for sex, age group, literacy, religion, ethnicity, health status, and social capital, with a random effect for cluster.

† Adjusted RR for the linear term for the potential mediator. Model 2 is a Poisson regression model of recent HIV testing on the mediators, with each mediator evaluated separately as the exposure in Models A to D. Models 2B to D include both a linear and quadratic term for the mediators. Analysis is stratified by study arm and adjusts for sex, age group, literacy, religion, ethnicity, health status, and social capital, with a robust standard error and random effect for cluster.

‡ Interaction p-value in Model 2A is for the study arm and the linear term for the potential mediator. Interaction p-values in Models 2B to D are for the study arm and the linear and quadratic terms for the mediators

In sub-group analysis, there was some evidence of an intervention effect among women for social cohesion (adjusted MD 0.17, 95% CI -0.01 to 0.35, p = 0.06), shared HIV concern (adjusted MD 0.16, 95% CI 0.00 to 0.31, p = 0.05), and critical consciousness (adjusted MD 0.18, 95% CI -0.02 to 0.37, p = 0.07). There was no evidence of an intervention effect among men (Table C in S1 Text). In older adults, weak evidence of an intervention effect was observed for social cohesion (adjusted MD 0.15, 95% CI -0.01 to 0.31, p = 0.06; Table E in S1 Text). Differences between study arms were not detected in adolescents.

### Effect of the potential mediators on outcome

**Fig 2:**
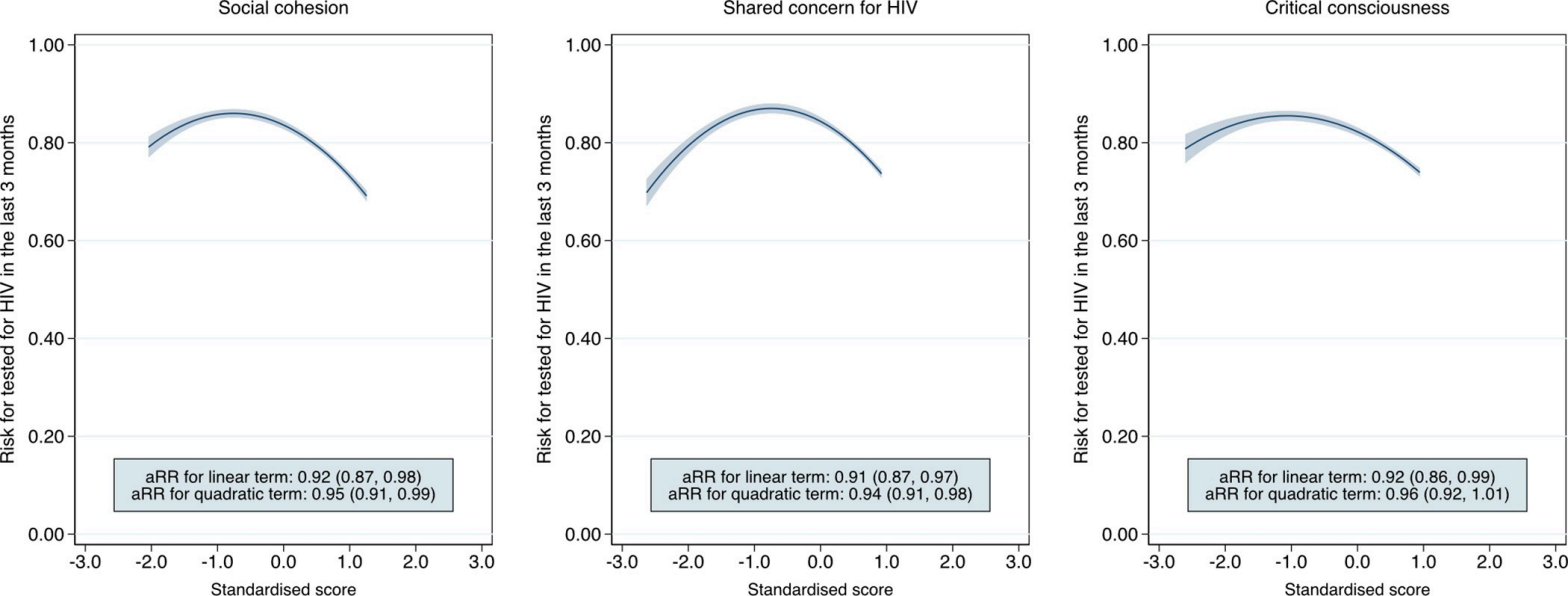
Prediction plots of recent HIV testing and potential mediators. aRR, adjusted risk ratio. Prediction plots with fitted values and 95% CIs. Prediction values obtained from Poisson regression of recent HIV testing on the linear and quadratic terms for the potential mediators, as standardised scores, in the community-led HIV self-testing arm. Fitted values obtained from a quadratic model of prediction values.

Estimates of causal associations between the standardised scores for the potential mediators and the outcome by study arm are presented in Table 2, with the risk ratio [RR] denoting the change in recent HIV testing (in the last 3 months) associated with a standard deviation increase in the score for the potential mediator. As illustrated in Fig 2, social cohesion and shared concern for HIV demonstrated a strong quadratic association with recent testing in the community-led HIVST arm, with a positive relationship measured at lower levels of scores followed by a waning effect at higher levels. Similarly, critical consciousness showed a positive association with recent testing at lower ranges of scores and a negative association at higher ranges. There was no evidence of an association between community HIV stigma and recent testing. In the SOC arm, there was no evidence for a strong association between each potential mediator and recent testing nor an interaction effect by study arm.

In sub-group analysis, community HIV stigma was strongly associated with recent HIV testing among women in the community-led HIVST arm (adjusted RR: 0.95, 95% CI 0.90 to 1.00, p = 0.05). Social cohesion also showed a strong quadratic relationship with recent testing (Fig C1 in S1 Text). Among men, shared HIV concern and critical consciousness were found to have a strong quadratic association with recent testing in the community-led HIVST arm (Fig C2 in S1 Text). There was also evidence of a quadratic relationship between shared HIV concern and recent testing among older adults in the community-led HIVST arm. No evidence of an association was observed between potential mediators and recent testing in adolescent counterparts (Fig D1 and D2 in S1 Text). Further, sub-group analysis did not detect a strong association between nearly all potential mediators and recent testing in the SOC arm as well as an interaction effect by study arm (Tables C and E in S1 Text).

### Direct and indirect effects of intervention on outcome

Analyses reported strong evidence for a direct effect of the community-led HIVST intervention on recent HIV testing (Table 3). Indirect effects appeared to be limited across potential mediators, overall and for most sub-groups (Tables D and F in S1 Text).

**Table 3:** Direct and indirect effect of community-led HIV self-testing intervention.

		Effect of intervention on HIV testing in the last 3 months		
		Direct effect	Indirect effect	Total effect
		Adjusted risk ratio (bootstrap CI)	Adjusted risk ratio (bootstrap CI)	Adjusted risk ratio (bootstrap CI)
(A)	Community HIV stigma	1.85 (1.72, 2.01)	1.00 (1.00, 1.01)	1.85 (1.72, 2.02)
(B)	Social cohesion *	1.75 (1.58, 1.99)	1.00 (0.99, 1.00)	1.74 (1.57, 1.98)
(C)	Shared concern for HIV *	1.79 (1.62, 2.02)	1.00 (0.99, 1.00)	1.78 (1.61, 2.01)
(D)	Critical consciousness *	1.75 (1.57, 1.97)	1.00 (0.99, 1.01)	1.75 (1.57, 1.97)

N = 1893. Estimates for direct and indirect effects are based on Models 1 and 3. Model 3 is a Poisson regression model of recent HIV testing on the study arm, with each potential mediator evaluated separately as a covariate in Models A to D. An interaction term for the study arm and the mediator is included. Analysis adjusts for sex, age group, literacy, religion, ethnicity, health status, and social capital, with a robust standard error and random effect for cluster. Confidence intervals are calculated using a bias-corrected bootstrap approach.

* Model includes log transformation of the potential mediator.

## Discussion

This study used causal mediation approaches to assess whether measures of community mobilisation and community HIV stigma mediated the effect of community-led delivery of HIVST on recent HIV testing. We found that the community-led HIVST intervention increased uptake of recent testing, with the effect appearing to be almost entirely direct. There was no evidence of indirect effects from changes in perceived social cohesion, shared HIV concern, critical consciousness, and community HIV stigma at the individual level. However, the intervention did slightly increase levels of perceived social cohesion and shared concern for HIV. Higher perceived social cohesion, community concern for HIV, and critical consciousness also apparently had a positive relationship with recent testing at lower levels of scores followed by a diminishing effect in the community-led HIVST arm. We found no evidence of intervention effects on perceptions of critical consciousness and community HIV stigma as well as an association between community stigma and recent testing. Few studies have quantitatively assessed mechanisms underlying the effect of community participation in health interventions. We conclude that the intervention effect mostly operated directly through community-driven service delivery of a novel HIV technology rather than through intermediate effects on individual perceptions of community mobilisation and HIV stigma.

We reported that the effect of the community-led HIVST intervention on recent HIV testing mostly occurred through direct pathways. Therefore, we mainly attribute the impact of the intervention to community ownership in the design and implementation of the HIVST campaign, which showed good coverage, rather than to changes in individual perceptions of social cohesion, shared HIV concern, and critical consciousness [20, 38]. The absence of indirect effects potentially stems from the intervention design. The intervention was developed for communities to periodically lead provision of HIV programmes, with frequency dependent on contextual factors including prevalence of undiagnosed HIV. The short implementation period had certain advantages, with the intervention yielding low unit costs for a community HIV testing programme [39]. However, such a strategy is perhaps more conducive to community participation in biomedical interventions in contrast with interventions aimed at impacting social and structural determinants of HIV. Previous studies of community participation in HIV programmes involved multi-year implementation to build community empowerment [9, 40, 41]. Longer implementation periods and more explicit intervention on dimensions of community empowerment may therefore be needed in order to influence downstream determinants of HIV but would likely require additional economic investment.

Despite the lack of evidence for indirect effects, we found that the community-led HIVST intervention may have led to changes in individual perceptions of shared HIV concern and social cohesion, overall and among sub-groups including women. Of the potential mediators, we posited that the intervention would most likely impact community HIV concern, which captures the importance of HIV as a collective priority, since the measure was specific to HIV. More generic scores included social cohesion, which captured community connectedness, and critical consciousness, which measured collective problem resolution. In the community-led HIVST arm, individual perceptions of social cohesion, community concern for HIV, and critical consciousness demonstrated a positive association with recent HIV testing at lower ranges of each score followed by a negative association at higher levels. The quadratic relationship may indicate the limited effect of community mobilisation domains on the outcome, which reaches a maximum point at low scores. A limited number of studies have quantitatively evaluated the contribution of community participation towards improving HIV-related outcomes. A multi-country study in southern Africa and Thailand reported that community mobilisation delivered with mobile HTS increased positive social norms for HIV testing [9]. Success was attributed to community engagement and relationship-building and context-specific, iterative implementation [42]. In South Africa, community mobilisation domains were associated with HIV testing following the implementation of a community mobilisation intervention [41], with interpersonal and community-level respect, communication, and empathy concluded to be integral components of change [43]. Our study adds to the literature by evaluating the role of community participation and continues to highlight the potential of investing in community health systems as a strategy for HIV prevention.

We hypothesised that the community-led HIVST intervention could reduce perceived community HIV stigma at the individual level by mobilising community support for HIV prevention or normalising HIV testing through HIVST. This study did not find strong evidence for an intervention effect on community HIV stigma nor an effect of community stigma levels on recent HIV testing. To reduce community HIV stigma, interventions would also likely require a longer period of implementation that specifically targeted drivers of stigma [44]. Disentangling the effects of HIV stigma can be challenging and is perhaps limited by our mediation framework. Community HIV stigma was posited to be on the causal pathway between the intervention and outcome, but it is possible, for example, that changes in community concern for HIV might first be necessary to reduce stigma. Further, community HIV stigma may have a bidirectional relationship with the outcome, with reduced stigma increasing uptake of HIVST and further normalising HIV testing and reducing stigma. In the context of a multiple component intervention with simultaneous multilevel impacts, the challenge of establishing causal effects could be addressed by prospectively measuring variables at sequential timepoints [15].

A strength of our study is the use of recent mediation methods to evaluate mechanisms of action underlying the effect of a complex intervention and their relative contribution to changes in the outcome. We used statistical techniques that extend traditional mediation approaches to allow for a binary outcome and intervention-mediator interaction. We also assessed mediation effects within a cluster-randomised design. By randomising the intervention, the study design minimises confounding and accounts for temporality assumptions between the intervention and mediator and the intervention and outcome, satisfying certain conditions important for causal interpretation [15]. Further, lessons from our study can potentially be applied to self-care interventions that involve and empower community groups in similar settings.

A limitation of our study is the use of a cross-sectional survey to measure the outcome, potential mediators, and mediator-outcome confounders, meaning the assumption that the mediator precedes the outcome was not automatically satisfied by the study design. For example, it is possible that engaging in HIV testing might affect an individual’s perception of shared HIV concern or community HIV stigma. To account for the direction of causality, we ideally would have measured the potential mediators and outcome in temporal order. The assumption that the intervention does not impact mediator-outcome confounders may also not be completely satisfied, though we aimed to select variables that conceptually were less likely be affected by the intervention. We also did not measure the potential mediators prior to the intervention and adjust for their levels at baseline, which may be a source of unmeasured mediator-outcome confounding. Sensitivity analysis can account for the respective associations between unmeasured confounders and the mediator and outcome and their impact on effect estimates [45]. We reported a lack of association between the potential mediators and outcome in the SOC arm, which could function as a proxy for baseline estimates and indicates that our conclusions would be unlikely to change.

Our mediation framework also assessed a single mediator variable at a time but did not evaluate direct and indirect effects based on a combined set of mediators [46]. Given that we did not find evidence of an indirect effect for each mediator, we would be unlikely to observe a combined effect. We also did not account for whether the potential mediators affected other mediators of interest on the causal pathway [46], including the possibility that changes in community mobilisation domains might be requisite for changes in community HIV stigma. Final limitations concern the measurement of outcomes and potential mediators. Measures for community mobilisation and community HIV stigma were based on perceived rather than experienced constructs and represent individual perceptions within the community [28]. We also only used a subset of domains of community mobilisation from a previously validated score [28]. Finally, our data were self-reported, which may have resulted in overestimation of outcomes and mediators in the community-led HIVST arm due to recall or social desirability bias.

Community-led delivery of HIVST increased uptake of recent HIV testing, with the intervention effect predominantly occurring through direct pathways rather than indirectly by modifying individual perceptions of community mobilisation and community HIV stigma. The community-led HIVST intervention apparently increased perceived shared concern for HIV and social cohesion, which alongside perceived critical consciousness had a protective effect on recent testing in the intervention arm but only at lower ranges of scores. By investigating mediation effects, we were able to evaluate factors important for optimising community-led HIV interventions. Our findings suggest that the impact of the community-led HIVST intervention mainly stemmed from community-driven service delivery rather than by modifying social and structural determinants of HIV. More frequent or active community participation might be required to achieve changes in community mobilisation and other social enablers as mechanisms for improving HIV-related outcomes. Trade-offs between immediate economic costs and building more sustainable community responses for HIV prevention, however, would need to be considered.

## Supporting information

S1 Text (DOCX)

S2 Text Inclusivity in global research. (DOCX)
