# Supplementary material for: Understanding mechanisms of impact from community-led delivery of HIV self-testing: Mediation analysis of a cluster-randomised trial in Malawi
Source: PLOS Glob Public Health. 2022 Oct 27;2(10):e0001129. doi: 10.1371/journal.pgph.0001129 (PMC10021599; doi:10.1371/journal.pgph.0001129)

**Supporting information**

**Table of Contents**

[Text A. Question items for community HIV stigma, community mobilisation, and social capital measures 2](#_Toc113275473)

[Table B. Implementation and process outcomes of community-led HIV self-testing intervention 4](#_Toc113275474)

[Table C. Effect of community-led HIV self-testing intervention and potential mediators by sex 7](#_Toc113275475)

[Table D. Direct and indirect effect of community-led HIV self-testing intervention by sex 9](#_Toc113275476)

[Table E. Effect of community-led HIV self-testing intervention and potential mediators by age group 10](#_Toc113275477)

[Table F. Direct and indirect effect of community-led HIV self-testing intervention by age group 12](#_Toc113275478)

[Fig A. Trial flow diagram 13](#_Toc113275479)

[Fig B. Process outcomes of community-led HIV self-testing intervention by sex and age group 14](#_Toc113275480)

[Fig C1. Prediction plots of recent HIV testing and potential mediators among women 15](#_Toc113275481)

[Fig C2. Prediction plots of recent HIV testing and potential mediators among men 16](#_Toc113275482)

[Fig D1. Prediction plots of recent HIV testing and potential mediators among adolescents 17](#_Toc113275483)

[Fig D2. Prediction plots of recent HIV testing and potential mediators among older adults 18](#_Toc113275484)

# Text A. Question items for community HIV stigma, community mobilisation, and social capital measures

| **Item** |
| --- |
| **Social cohesion *** |
| For each of the following statements, please indicate whether you strongly agree, somewhat agree or disagree. |
| 1. People in this village are willing to help their neighbors. |
| 2. This is a close-knit community. |
| 3. People in this village can be trusted. |
| 4. People in this village generally get along well with each other. |
| 5. People in this village share the same values. |
| 6. People in this village look out for each other. |
| **Shared concern for HIV** * |
| For each of the following statements, please indicate whether you strongly agree, somewhat agree or disagree. |
| 1. People in your village are concerned about HIV. |
| 2. People in your village consider HIV/AIDS an important issue. |
| 3. People in your village talk openly about HIV. |
| 4. People in your village believe that HIV impacts the community. |
| 5. People in your village talk about HIV/AIDS at community meetings. |
| 6. People in your village work together to prevent HIV from spreading. |
| 7. People in your village work together to reduce the effects of HIV. |
| 8. People in your village believe they can change the course of the HIV/AIDS epidemic. |
| 9. People in your village exchange information about HIV/AIDS. |
| 10. People in your village take HIV/AIDS seriously. |
| **Critical consciousness** * |
| For each of the following statements, please indicate whether you strongly agree, somewhat agree or disagree. |
| 1. People work together to solve problems in the village. |
| 2. People in your village talk to each other about how to solve village problems. |
| 3. People in your village enjoy discussing different ways to solve village problems. |
| 4. People in your village are open to hearing different views about community problems and solutions. |
| 5. People in your village volunteer to help solve village problems. |
| 6. People in your village think about why there are problems so they can address the cause of problems. |
| 7. There is a lot of cooperation between groups in the village. |
| 8. People in this village not only talk about problems but they also try to solve them. |
| 9. If your community fails to resolve a community problem, they will try another different approach to solve the problem. |
| 10. If your community fails to resolve a community problem, they will learn from that experience and do a better job when they try to solve the problem in the future. |
| 11. If leaders in the village fail to resolve a village problem, the villagers will work together to find a solution. |
| **Community HIV stigma** † |
| For each of the following statements, please indicate whether you strongly agree, somewhat agree or disagree. |
| 1. People living with or thought to be living with HIV are sometimes physically assaulted. |
| 2. People sometimes talk badly about people living with or thought to be living with HIV. |
| 3. People living with or thought to be living with HIV lose respect or standing. |
| 4. People living with or thought to be living with HIV are verbally insulted, harassed, and/or threatened. |
| **Social capital** ‡ |
| Are you a member of any of the following committees or groups? |
| 1. Chiefs council |
| 2. Development committee |
| 3. Health committee |
| 4. School committee |
| 5. Women's group |
| 6. Peer/youth group |
| 7. Celebration/burial group |
| 8. Commerce/finance group |
| 9. Church or mosque |
| 10. Sports group |

* Questions were adapted from Lippman et al. [28]. Responses used a three-point Likert scale (0-2).

† Questions were adapted from Stangl et al. [29]. Responses used a three-point Likert scale (0-2).

† Questions were adapted from the Malawi Longitudinal Study of Families and Health [https://malawi.pop.upenn.edu/]. Responses were binary (0-1).

# Table B. Implementation and process outcomes of community-led HIV self-testing intervention

| **Cluster** | **Description of strategies** | **Description of community health volunteers** |  | **Post-intervention survey** | | |
| --- | --- | --- | --- | --- | --- | --- |
|  |  |  | **Kits distributed** |  | **Heard of self-testing for HIV** | **Self-tested for HIV in the last 3 months** |
|  |  |  | **N** | **N** | **n (%)** | **n (%)** |
| 1 | Sensitisation and distribution: community meeting, door-to-door, bawo match, boreholes, religious centres.  Linkage: active follow-up, accompany to facility. | 9 CHAG members: women (n=5), men (n=4); 20-39 years (n=8), ≥40 years (n=1).  12 CVs*: women (n=3), men (n=9); 20-39 years (n=10), ≥40 years (n=1). | 1062 | 68 | 66 (97.1%) | 63 (92.6%) |
| 2 | Sensitisation and distribution: community meeting, door-to-door, community hall, religious centres, sports fields, community hall.  Linkage: active follow-up, material assistance, phone referral. | 10 CHAG members: women (n=4), men (n=6); 15-19 years (n=1), 20-39 years (n=5), ≥40 years (n=4).  15 CVs*: women (n=8), men (n=7); 20-39 years (n=14). | 2218 | 77 | 77 (100.0%) | 62 (80.5%) |
| 3 | Sensitisation and distribution: community meeting, door-to-door, bawo match, health post, markets, religious centres.  Linkage: active follow-up, accompany to facility, material assistance. | 10 CHAG members: women (n=5), men (n=5); 20-39 years (n=9), ≥40 years (n=1).  16 CVs: women (n=8), men (n=8); 15-19 years (n=2), 20-39 years (n=12), ≥40 years (n=2). | 1621 | 49 | 48 (98.0%) | 36 (73.5%) |
| 4 | Sensitisation and distribution: community meeting, door-to-door, agricultural fields, markets, religious centres, schools, sports fields, video shows.  Linkage: active follow-up, material assistance, phone referral. | 11 CHAG members: women (n=9), men (n=2); 20-39 years (n=7), ≥40 years (n=4).  14 CVs*: women (n=11), men (n=2); 20-39 years (n=11), ≥40 years (n=2). | 1806 | 43 | 41 (95.3%) | 25 (58.1%) |
| 5 | Sensitisation and distribution: community meeting, door-to-door, bawo match, bicycle repair shops, boreholes, fishing docks, markets, restaurants and bars, schools, sports fields, video shows.  Linkage: accompany to facility, material assistance. | 10 CHAG members: women (n=6), men (n=4); 15-19 years (n=1), 20-39 years (n=8), ≥40 years (n=1).  10 CVs: women (n=3), men (n=7); 15-19 years (n=2), 20-39 years (n=7), ≥40 years (n=1). | 1698 | 55 | 54 (98.2%) | 44 (80.0%) |
| 6 | Sensitisation and distribution: community meeting, businesses and shops, door-to-door, markets, religious centres, schools, sports fields.  Linkage: active follow-up, accompany to facility. | 11 CHAG members*: women (n=5), men (n=6); 20-39 years (n=9), ≥40 years (n=1).  20 CVs: women (n=12), men (n=8); 20-39 years (n=17), ≥40 years (n=3). | 2864 | 67 | 64 (95.5%) | 27 (40.3%) |
| 7 | Sensitisation and distribution: community meeting and gule wamkulu, door-to-door, markets, religious centres.  Linkage: active follow-up, material assistance, phone referral. | 11 CHAG members: women (n=6), men (n=5); 20-39 years (n=11).  11 CVs: women (n=5), men (n=6); 20-39 years (n=11). | 2379 | 95 | 92 (96.8%) | 74 (77.9%) |
| 8 | Sensitisation and distribution: community meeting, door-to-door, barbershops, bawo match, boreholes, maize mills, under-five clinic.  Linkage: active follow-up, accompany to facility, material assistance. | 11 CHAG members: women (n=8), men (n=3); 15-19 years (n=1), 20-39 years (n=9), ≥40 years (n=1). 16 CVs: women (n=8), men (n=8); 15-19 years (n=1), 20-39 years (n=15). | 984 | 52 | 52 (100.0%) | 44 (84.6%) |
| 9 | Sensitisation and distribution: community meeting, door-to-door, bawo match, boreholes, businesses and shops, maize mills, religious centres, schools, sports fields, video shows, youth clubs.  Linkage: active follow-up, material assistance, phone referral. | 11 CHAG members: women (n=5), men (n=6); 20-39 years (n=5), ≥40 years (n=6). 13 CVs: women (n=8), men (n=5); 15-19 years (n=1), 20-39 years (n=10), ≥40 years (n=2). | 1596 | 55 | 55 (100.0%) | 45 (81.8%) |
| 10 | Sensitisation and distribution: community meeting, door-to-door, bawo match, boreholes, businesses and shops, community hall, markets, schools, sports fields. | 7 CHAG members*: women (n=4), men (n=3); 20-39 years (n=2), ≥40 years (n=3). 10 CVs: women (n=4), men (n=6), 20-39 years (n=5), ≥40 years (n=5). | 819 | 60 | 57 (95.0%) | 49 (81.7%) |
| 11 | Sensitisation and distribution: community meeting, door-to-door, agricultural fields, community hall, markets, religious centres, schools, sports fields, youth hall.  Linkage: active follow-up, phone referral. | 12 CHAG members: women (n=11), men (n=1); 20-39 years (n=10), ≥40 years (n=2). 16 CVs: women (n=13), men (n=3); 20-39 years (n=14), ≥40 years (n=2). | 2490 | 79 | 66 (83.5%) | 36 (45.6%) |
| 12 | Sensitisation and distribution: community meeting, door-to-door, chief home, community hall, sports fields, video shows.  Linkage: active follow-up, accompany to facility. | 11 CHAG members*: women (n=7), men (n=4); 20-39 years (n=7), ≥40 years (n=3). 8 CVs: women (n=6), men (n=2); 20-39 years (n=7), ≥40 years (n=1). | 1038 | 97 | 90 (92.8%) | 64 (66.0%) |
| 13 | Sensitisation and distribution: community meeting, door-to-door, bawo match, fishing docks.  Linkage: material assistance, phone referral. | 11 CHAG members: women (n=4), men (n=7); 20-39 years (n=9), ≥40 years (n=2). 8 CVs*: women (n=2), men (n=5); 20-39 years (n=4), ≥40 years (n=3). | 947 | 53 | 53 (100.0%) | 41 (77.4%) |
| 14 | Sensitisation and distribution: community meeting, door-to-door, boreholes, health post, markets, religious centres, sports fields.  Linkage: active follow-up, accompany to facility, material assistance. | 10 CHAG members: women (n=9), men (n=1); 20-39 years (n=10). 13 CVs: women (n=7), men (n=6); 15-19 years (n=2), 20-39 years (n=10), ≥40 years (n=1). | 1010 | 54 | 54 (100.0%) | 45 (83.3%) |
| 15 | Sensitisation and distribution: community meeting, door-to-door, community hall, markets, religious centres, sports fields.  Linkage: active follow-up, accompany to facility, material assistance. | 12 CHAG members: women (n=9), men (n=3); 20-39 years (n=9), ≥40 years (n=3). 16 CVs*: women (n=9), men (n=6); 20-39 years (n=11), ≥40 years (n=4). | 1784 | 66 | 63 (95.5%) | 49 (74.2%) |
| Total |  |  | 24316 | 970 | 932 (96.1%) | 704 (72.6%) |

CHAG, community health action group; CV, community volunteer.

* Missing data on sex or age.

# Table C. Effect of community-led HIV self-testing intervention and potential mediators by sex

|  | | **(1)** | **(2)** | | |
| --- | --- | --- | --- | --- | --- |
|  |  | **Effect of intervention on potential mediator *** | **Effect of potential mediator on HIV testing in the last 3 months**  **by study arm †** | | |
|  |  |  | **Community-led HIVST** | **SOC** | **p-value for interaction for study arm ‡** |
|  |  | **Adjusted mean difference (95% CI)** | **Adjusted risk ratio  (95% CI)** | **Adjusted risk ratio  (95% CI)** |  |
|  |  | **p-value** | **p-value** | **p-value** |  |
| **Female** | | | | | |
| (A) | Community HIV stigma | -0.01 (-0.21, 0.19)  0.91 | 0.95 (0.90, 1.00)  0.05 | 0.92 (0.81, 1.04)  0.18 | 0.80 |
| (B) | Social cohesion | 0.17 (-0.01, 0.35)  0.06 | 0.95 (0.89, 1.01)  0.09 | 1.00 (0.93, 1.07)  0.99 | 0.27 |
|  | Social cohesion^2^ |  | 0.96 (0.94, 0.99)  0.01 | 1.00 (0.95, 1.05)  1.00 |  |
| (C) | Shared concern for HIV | 0.16 (0.00, 0.31)  0.05 | 0.93 (0.88, 0.99)  0.02 | 0.94 (0.84, 1.06)  0.32 | 0.67 |
|  | Shared concern for HIV^2^ |  | 0.98 (0.93, 1.02)  0.26 | 0.95 (0.88, 1.02)  0.17 |  |
| (D) | Critical consciousness | 0.18 (-0.02, 0.37)  0.07 | 0.95 (0.88, 1.03)  0.22 | 1.00 (0.89, 1.13)  0.98 | 0.66 |
|  | Critical consciousness^2^ |  | 0.99 (0.95, 1.02)  0.45 | 1.01 (0.91, 1.11)  0.91 |  |
| **Male** | | | | | |
| (A) | Community HIV stigma | 0.00 (-0.17, 0.17)  1.00 | 0.99 (0.93, 1.05)  0.68 | 0.92 (0.78, 1.08)  0.31 | 0.33 |
| (B) | Social cohesion | 0.10 (-0.09, 0.29)  0.30 | 0.89 (0.83, 0.96)  0.003 | 1.06 (0.93, 1.20)  0.39 | 0.10 |
|  | Social cohesion^2^ |  | 0.94 (0.87, 1.03)  0.18 | 1.01 (0.88, 1.15)  0.92 |  |
| (C) | Shared concern for HIV | 0.11 (-0.08, 0.30)  0.24 | 0.89 (0.82, 0.97)  0.01 | 1.04 (0.81, 1.32)  0.78 | 0.18 |
|  | Shared concern for HIV^2^ |  | 0.89 (0.82, 0.97)  0.006 | 1.04 (0.90, 1.21)  0.61 |  |
| (D) | Critical consciousness | 0.01 (-0.19, 0.21)  0.89 | 0.88 (0.82, 0.95)  0.001 | 1.06 (0.86, 1.29)  0.59 | 0.12 |
|  | Critical consciousness^2^ |  | 0.92 (0.86, 0 .99)  0.04 | 1.00 (0.84, 1.19)  0.98 |  |

C, control; HIVST, HIV self-testing; I, intervention; SOC, standard of care. Female, N=1136; Male, N=757.

* Adjusted mean difference for the study arm (I-C). Model 1 is a linear regression model of the potential mediators on the study arm, with each mediator evaluated separately as the outcome in Models A to D. Analysis adjusts for age group, literacy, religion, ethnicity, health status, and social capital, with a random effect for cluster.

† Adjusted RR for the linear term for the potential mediator. Model 2 is a Poisson regression model of recent HIV testing on the mediators with each mediator evaluated separately as the exposure in Models A to D. Models 2B to D include both a linear and quadratic term for the mediators. Analysis is stratified by study arm and adjusts for age group, literacy, religion, ethnicity, health status, and social capital, with a robust standard error and random effect for cluster.

‡ Interaction p-value in Model 2A is for the study arm and the linear term for the potential mediator. Interaction p-values in Models 2B to D are for the study arm and the linear and quadratic terms for the mediators.

# Table D. Direct and indirect effect of community-led HIV self-testing intervention by sex

|  | | **Effect of intervention on HIV testing in the last 3 months** | | |
| --- | --- | --- | --- | --- |
|  | | **Direct effect** | **Indirect effect** | **Total effect** |
|  |  | **Adjusted risk ratio  (bootstrap CI)** | **Adjusted risk ratio  (bootstrap CI)** | **Adjusted risk ratio  (bootstrap CI)** |
| **Female** | | | | |
| (A) | Community HIV stigma | 1.80 (1.66, 2.00) | 1.00 (0.99, 1.01) | 1.81 (1.66, 2.00) |
| (B) | Social cohesion * | 1.75 (1.51, 2.02) | 1.00 (0.99, 1.01) | 1.75 (1.52, 2.02) |
| (C) | Shared concern for HIV * | 1.75 (1.54, 2.01) | 0.99 (0.98, 1.00) | 1.74 (1.53, 1.99) |
| (D) | Critical consciousness * | 1.72 (1.52, 1.99) | 1.00 (0.98, 1.00) | 1.71 (1.52, 1.99) |
| **Male** | | | | |
| (A) | Community HIV stigma | 1.92 (1.67, 2.25) | 1.00 (0.99, 1.01) | 1.92 (1.67, 2.24) |
| (B) | Social cohesion * | 1.72 (1.45, 2.17) | 0.99 (0.97, 1.01) | 1.71 (1.45, 2.15) |
| (C) | Shared concern for HIV * | 1.82 (1.49, 2.22) | 1.00 (0.99, 1.03) | 1.83 (1.51, 2.24) |
| (D) | Critical consciousness * | 1.79 (1.49, 2.18) | 1.02 (0.99, 1.05) | 1.82 (1.53, 2.23) |

Female: N=1136; Male: N=757. Estimates for direct and indirect effects are based on Models 1 and 3. Model 3 is a Poisson regression model of recent HIV testing on the study arm, with each potential mediator evaluated separately as a covariate in Models A to D. An interaction term for the study arm and the mediator is included. Analysis adjusts for age group, literacy, religion, ethnicity, health status, and social capital, with a robust standard error and random effect for cluster. Confidence intervals are calculated using a bias-corrected bootstrap approach.

* Model includes log transformation of the potential mediator.

# Table E. Effect of community-led HIV self-testing intervention and potential mediators by age group

|  | | **(1)** | **(2)** | | |
| --- | --- | --- | --- | --- | --- |
|  |  | **Effect of intervention on potential mediator *** | **Effect of potential mediator on HIV testing in the last 3 months**  **by study arm †** | | |
|  |  |  | **Community-led HIVST** | **SOC** | **p-value for interaction for study arm ‡** |
|  |  | **Adjusted mean difference (95% CI)** | **Adjusted risk ratio  (95% CI)** | **Adjusted risk ratio  (95% CI)** |  |
|  |  | **p-value** | **p-value** | **p-value** |  |
| **15-19 years** | | | | | |
| (A) | Community HIV stigma | 0.05 (-0.14, 0.24)  0.58 | 0.95 (0.87, 1.03)  0.24 | 0.86 (0.72, 1.02)  0.08 | 0.18 |
| (B) | Social cohesion | -0.06 (-0.26, 0.13)  0.54 | 0.98 (0.90, 1.06)  0.57 | 1.01 (0.83, 1.23)  0.92 | 0.85 |
|  | Social cohesion^2^ |  | 0.95 (0.88, 1.02)  0.15 | 0.99 (0.83, 1.17)  0.88 |  |
| (C) | Shared concern for HIV | -0.02 (-0.23, 0.19)  0.84 | 0.92 (0.83, 1.01)  0.07 | 0.99 (0.83, 1.16)  0.87 | 0.47 |
|  | Shared concern for HIV^2^ |  | 0.95 (0.89, 1.02)  0.16 | 1.02 (0.90, 1.14)  0.79 |  |
| (D) | Critical consciousness | -0.08 (-0.27, 0.10)  0.36 | 0.94 (0.86, 1.02)  0.13 | 0.95 (0.82, 1.10)  0.52 | 0.74 |
|  | Critical consciousness^2^ |  | 0.94 (0.87, 1.01)  0.11 | 0.95 (0.79, 1.15)  0.63 |  |
| **≥40 years** | | | | | |
| (A) | Community HIV stigma | -0.06 (-0.30, 0.18)  0.64 | 0.93 (0.85, 1.02)  0.12 | 0.83 (0.70, 0.98)  0.03 | 0.25 |
| (B) | Social cohesion | 0.15 (-0.01, 0.31)  0.06 | 0.92 (0.86, 0.99)  0.02 | 1.10 (0.96, 1.26)  0.17 | 0.28 |
|  | Social cohesion^2^ |  | 0.98 (0.93, 1.03)  0.36 | 0.93 (0.79, 1.08)  0.34 |  |
| (C) | Shared concern for HIV | 0.10 (-0.10, 0.29)  0.33 | 0.91 (0.82, 1.00)  0.06 | 0.98 (0.81, 1.20)  0.88 | 0.69 |
|  | Shared concern for HIV^2^ |  | 0.85 (0.77, 0.95)  0.004 | 0.82 (0.63, 1.07)  0.14 |  |
| (D) | Critical consciousness | 0.13 (-0.13, 0.38)  0.33 | 0.91 (0.79, 1.04)  0.17 | 1.01 (0.81, 1.26)  0.93 | 0.76 |
|  | Critical consciousness^2^ |  | 0.94 (0.87, 1.03)  0.18 | 1.02 (0.88, 1.19)  0.78 |  |

C, control; HIVST, HIV self-testing; I, intervention; SOC, standard of care. 15-19 years, N=407; ≥40 years, N=532.

* Adjusted mean difference for the study arm (I-C). Model 1 is a linear regression model of the potential mediators on the study arm, with each mediator evaluated separately as the outcome in Models A to D. Analysis adjusts for sex, literacy, religion, ethnicity, health status, and social capital, with a random effect for cluster.

† Adjusted RR for the linear term for the potential mediator. Model 2 is a Poisson regression model of recent HIV testing on the mediators, with each mediator evaluated separately as the exposure in Models A to D. Models 2B to D include both a linear and quadratic term for the mediators. Analysis is stratified by study arm and adjusts for sex, literacy, religion, ethnicity, health status, and social capital, with a robust standard error and random effect for cluster.

‡ Interaction p-value in Model 2A is for the study arm and the linear term for the potential mediator. Interaction p-values in Models 2B to D are for the study arm and the linear and quadratic terms for the mediators.

# Table F. Direct and indirect effect of community-led HIV self-testing intervention by age group

|  | | **Effect of intervention on HIV testing in the last 3 months** | | |
| --- | --- | --- | --- | --- |
|  | | **Direct effect** | **Indirect effect** | **Total effect** |
|  |  | **Adjusted risk ratio  (bootstrap CI)** | **Adjusted risk ratio  (bootstrap CI)** | **Adjusted risk ratio  (bootstrap CI)** |
| **15-19 years** | | | | |
| (A) | Community HIV stigma | 1.77 (1.49, 2.12) | 1.00 (0.98, 1.01) | 1.76 (1.48, 2.11) |
| (B) | Social cohesion * | 1.66 (1.31, 2.13) | 1.00 (0.97, 1.02) | 1.66 (1.32, 2.13) |
| (C) | Shared concern for HIV * | 1.66 (1.22, 2.25) | 1.01 (0.99, 1.06) | 1.67 (1.24, 2.27) |
| (D) | Critical consciousness * | 1.61 (0.89, 2.50) | 1.05 (1.01, 1.12) | 1.69 (0.95, 2.59) |
| **≥40 years** | | | | |
| (A) | Community HIV stigma | 1.78 (1.56, 2.02) | 1.00 (1.00, 1.01) | 1.78 (1.56, 2.03) |
| (B) | Social cohesion * | 1.71 (1.43, 2.13) | 1.00 (0.98, 1.03) | 1.71 (1.44, 2.14) |
| (C) | Shared concern for HIV * | 1.71 (1.46, 2.12) | 1.00 (0.99, 1.02) | 1.71 (1.46, 2.12) |
| (D) | Critical consciousness * | 1.59 (1.34, 1.91) | 1.00 (1.00, 1.02) | 1.59 (1.34, 1.91) |

15-19 years, N=407; ≥40 years, N=532. Estimates for direct and indirect effects are based on Models 1 and 3. Model 3 is a Poisson regression model of recent HIV testing on the study arm, with each potential mediator evaluated separately as a covariate in Models A to D. An interaction term for the study arm and the mediator is included. Analysis adjusts for sex, literacy, religion, ethnicity, health status, and social capital, with a robust standard error and random effect for cluster. Confidence intervals are calculated using a bias-corrected bootstrap approach.

* Model includes log transformation of the potential mediator.

# Fig A. Trial flow diagram

Flow diagram of the cluster-randomised trial.


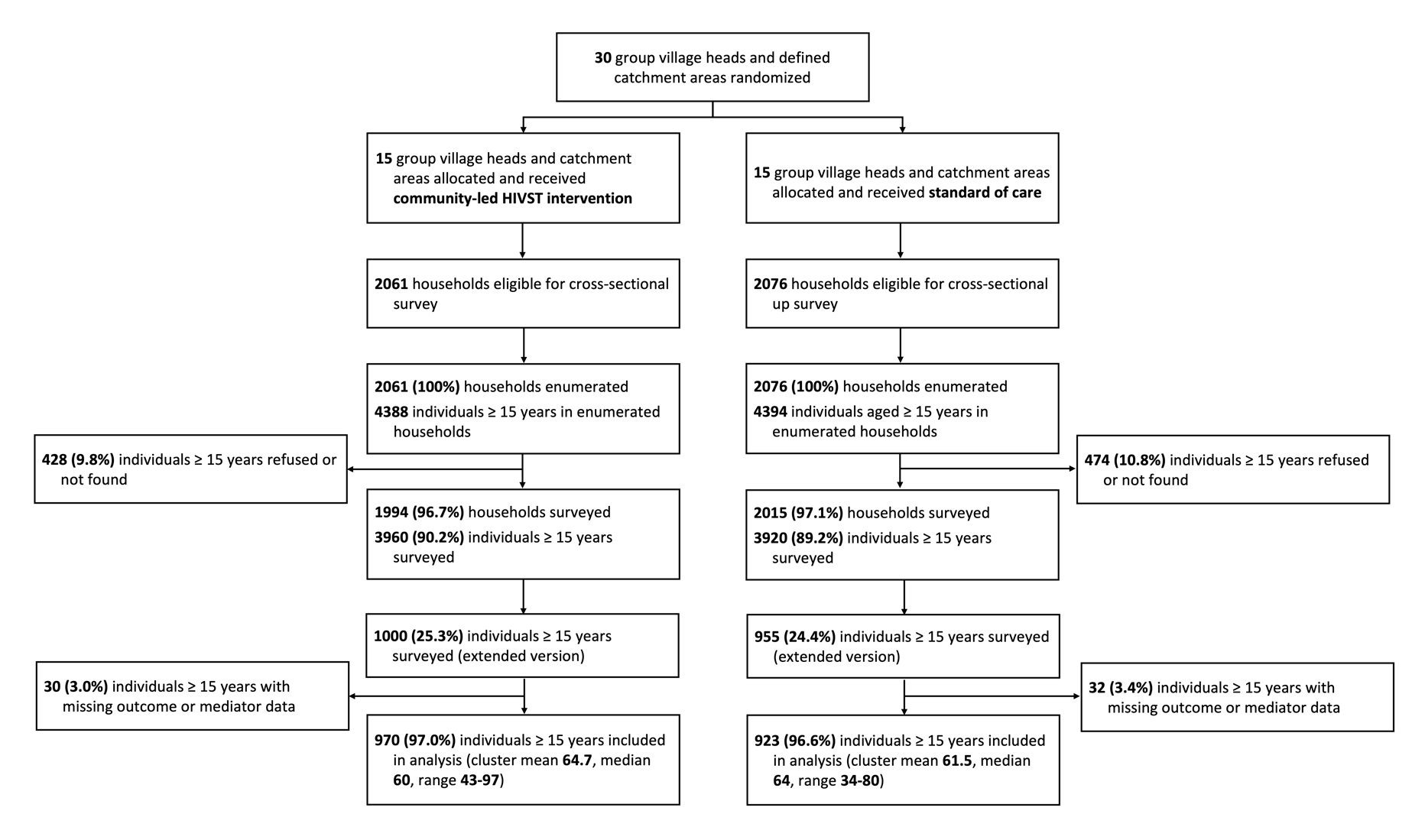


# Fig B. Process outcomes of community-led HIV self-testing intervention by sex and age group

HIVST, HIV self-testing; SOC, standard of care. Graphs indicate the proportions and 95% CIs adjusted for clustering following the community-led HIVST intervention. Data are stratified by study arm, sex, and age group.


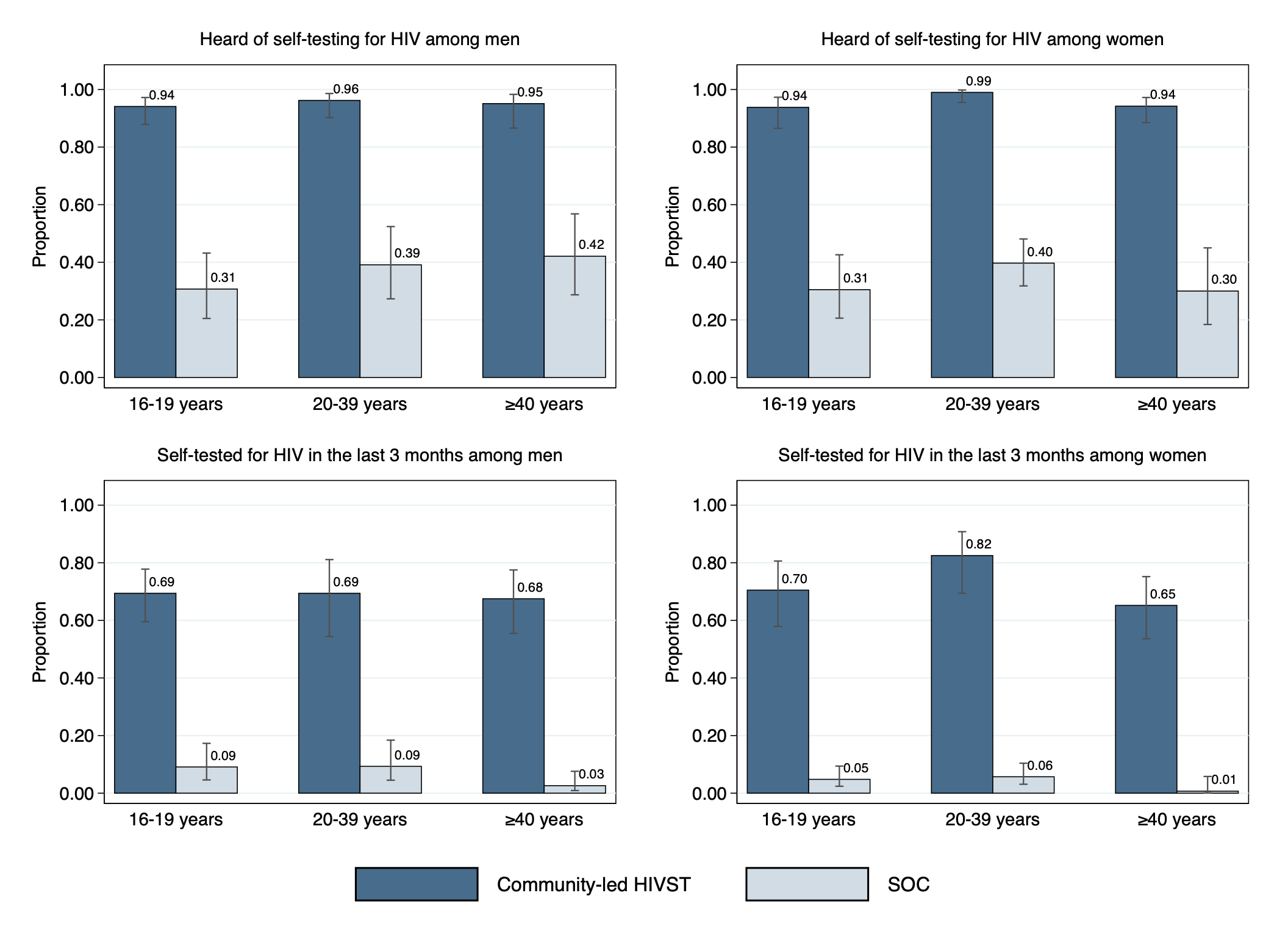


# Fig C1. Prediction plots of recent HIV testing and potential mediators among women

aRR, adjusted risk ratio.

Prediction plots with fitted values and 95% CIs. Prediction values obtained from Poisson regression of recent HIV testing on the linear and quadratic terms for the potential mediators among women in the community-led HIV self-testing arm. Scores for mediators are standardised. Fitted values obtained from a quadratic model of prediction values.


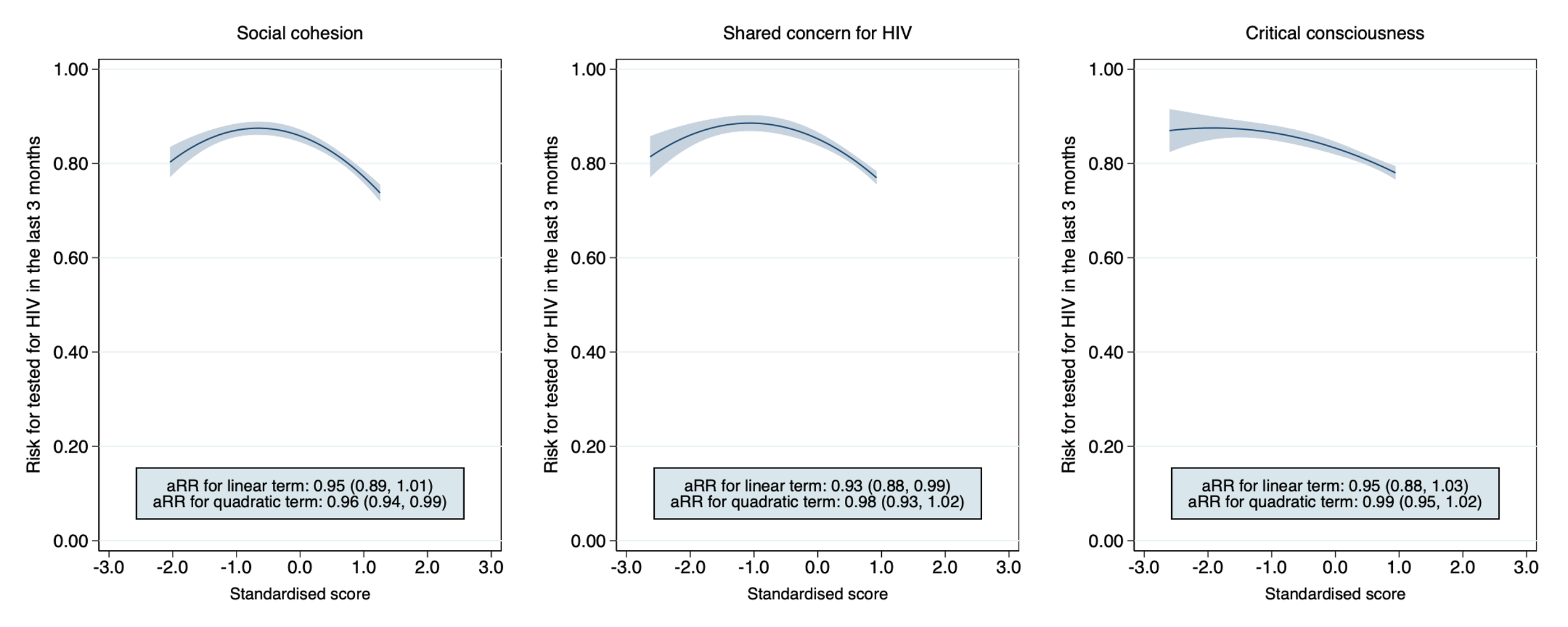


# Fig C2. Prediction plots of recent HIV testing and potential mediators among men

aRR, adjusted risk ratio.

Prediction plots with fitted values and 95% CIs. Prediction values obtained from Poisson regression of recent HIV testing on the linear and quadratic terms for the potential mediators among men in the community-led HIV self-testing arm. Scores for mediators are standardised. Fitted values obtained from a quadratic model of prediction values.


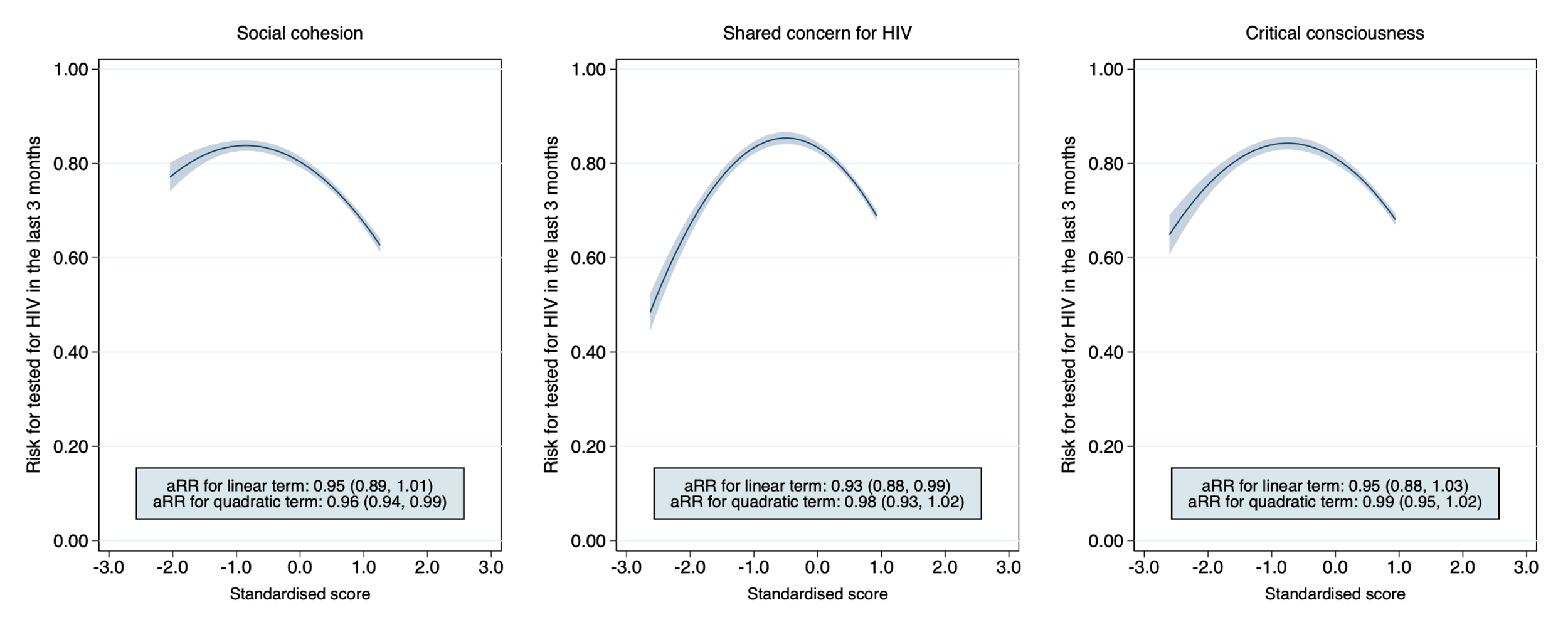


# Fig D1. Prediction plots of recent HIV testing and potential mediators among adolescents

aRR, adjusted risk ratio.

Prediction plots with fitted values and 95% CIs. Prediction values obtained from Poisson regression of recent HIV testing on the linear and quadratic terms for the potential mediators among adolescents 15-19 years in the community-led HIV self-testing arm. Scores for mediators are standardised. Fitted values obtained from a quadratic model of prediction values.


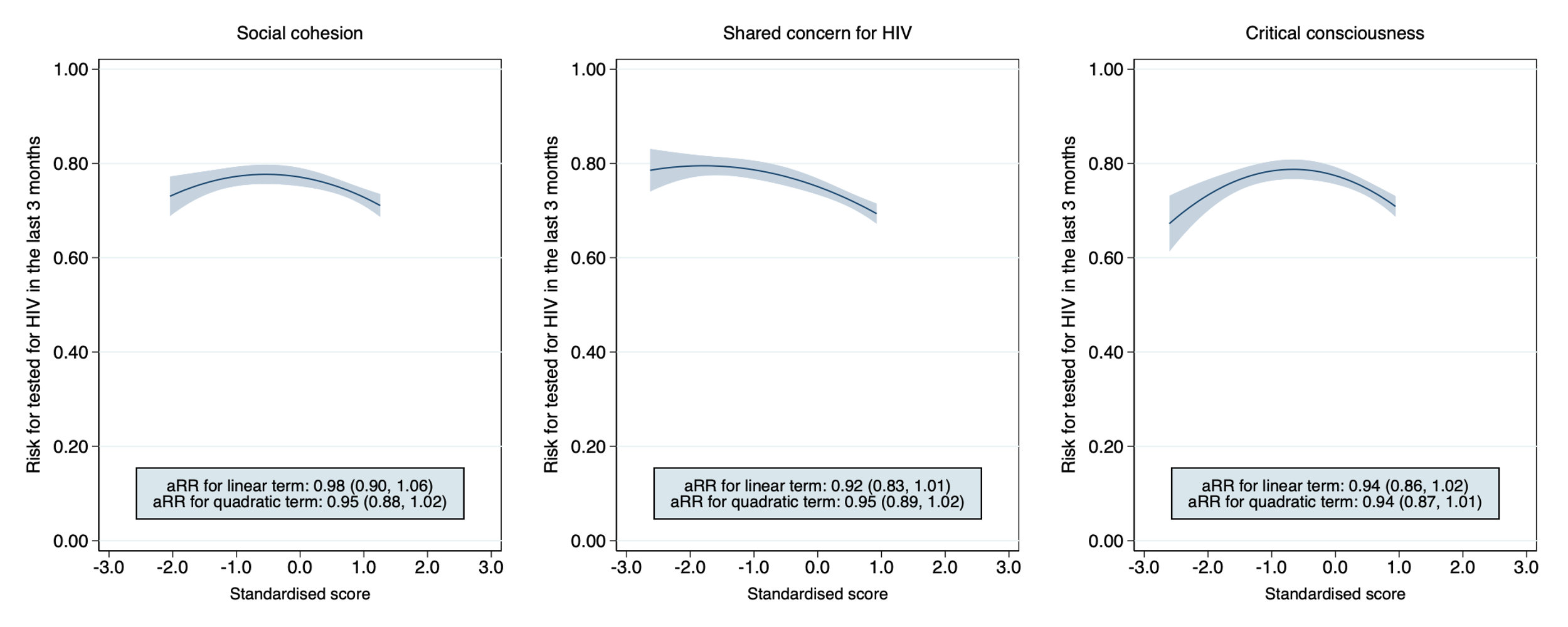


# Fig D2. Prediction plots of recent HIV testing and potential mediators among older adults

aRR, adjusted risk ratio.

Prediction plots with fitted values and 95% CIs. Prediction values obtained from Poisson regression of recent HIV testing on the linear and quadratic terms for the potential mediators among older adults 40 years and above in the community-led HIV self-testing arm. Scores for mediators are standardised. Fitted values obtained from a quadratic model of prediction values.


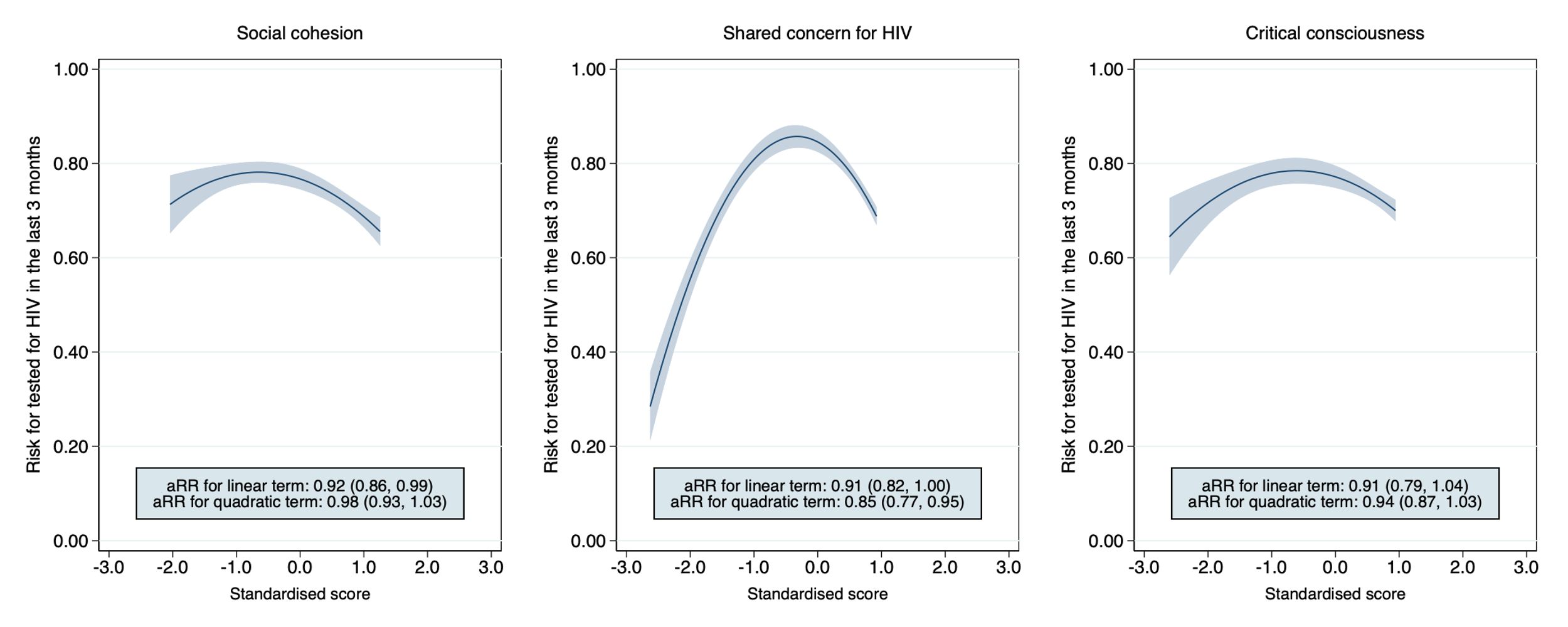

Supplement: S1 Text — (DOCX) [file pgph.0001129.s001.docx]
